# A Strategy for Anode Recovery and Upgrading by In Situ Growth of Iron-Based Oxides on Microwave-Puffed Graphite

**DOI:** 10.3390/molecules29133219

**Published:** 2024-07-07

**Authors:** Wenxin Chen, Jing Sun, Pingshan Jia, Wenlong Wang, Zhanlong Song, Ziliang Wang, Xiqiang Zhao, Yanpeng Mao

**Affiliations:** National Engineering Laboratory for Reducing Emissions from Coal Combustion, Engineering Research Center of Environmental Thermal Technology of Ministry of Education, Shandong Key Laboratory of Energy Carbon Reduction and Resource Utilization, School of Energy and Power Engineering, Shandong University, Jinan 250061, China

**Keywords:** lithium-ion batteries, spent graphite, transition metal oxides, graphite intercalation compounds, composite anode

## Abstract

Faced with the increasing volume of retired lithium-ion batteries (LIBs), recycling and reusing the spent graphite (SG) is of great significance for resource sustainability. Here, a facile method for transforming the SG into a carbon framework as well as loading Fe_2_O_3_ to form a composite anode with a sandwich structure is proposed. Taking advantage of the fact that the layer spacing of the spent graphite naturally expands, impurities and intercalants are eliminated through microwave thermal shock to produce microwave-puffed graphite (MPG) with a distinct three-dimensional structure. Based on the mechanism of microwave-induced gasification intercalation, a Fe_2_O_3_-MPG intercalation compound (Fe_2_O_3_-MPGIC) anode material was constructed by introducing iron precursors between the framework layers and subsequently converting them into Fe_2_O_3_ through annealing. The Fe_2_O_3_-MPGIC anode exhibits a high reversible capacity of 1000.6 mAh g^−1^ at 200 mA g^−1^ after 100 cycles and a good cycling stability of 504.4 mAh g^−1^ at 2000 mA g^−1^ after 500 cycles. This work can provide a reference for the feasible recycling of SG and development of high-performance anode materials for LIBs.

## 1. Introduction

As efficient energy storage devices in the burgeoning renewable energy sector, lithium-ion batteries (LIBs), have become the main power source for electric vehicles and hybrid electric vehicles, contributing significantly to the reduction in CO_2_ emissions. However, the expansion of LIBs manufacturing is accompanied by a growing demand for raw materials and a subsequent increase in retired batteries due to their limited lifespan. It is estimated that at least 650 GWh of LIBs will be scrapped in 2025 [1]. Improper disposal of spent batteries presents potential hazards such as explosions and pollutant emissions, and results in the squandering of valuable raw materials. Consequently, the recycling of spent batteries is imperative to safeguard the environment and ensure the sustainability of resources.

The recycling of cathode materials has received considerable attention due to their high metal content [2,3]. However, anode materials (e.g., graphite, hard carbon) are often overlooked or simply used as reducing agents [4]. Graphite, the most commonly used anode material, constitutes approximately 20% of the total mass of LIBs. Proper recycling of graphite is crucial for alleviating the graphite resource shortage and reducing the energy and costs associated with graphite mining and production.

Compared with natural graphite, spent graphite (SG) includes various metal and organic impurities that need to be removed. While SG can be regenerated through acid leaching [5] and pyrometallurgy [6] for reuse as anode material, this process is not attractive due to its chemical and energy consumption [7]. More importantly, the inherent low capacity of graphite cannot meet the demand for high-energy-density LIBs, and high-capacity anodes such as silicon [8] and metal oxides [9] have become the main focus of research.

Ferric oxide (Fe_2_O_3_), as one of the most promising alternatives to graphite anodes, offers advantages such as wide availability, low cost, high theoretical reversible specific capacity, and good safety. However, they suffer from volume expansion and low conductivity throughout the cycling process, which markedly affect cycling stability and rate performance. Compounding with carbon materials is one of the most common methods for enhancing Fe_2_O_3_ anodes. Guo et al. [10] synthesized uniform α-Fe_2_O_3_ nanoparticles with a narrow gap (~1.4 nm), which were immobilized on CNTs through N-doped carbon (α-Fe_2_O_3_/CNTs-NC). Chen et al. [11] chose to embed Fe_2_O_3_ nanoparticles within the shells of hollow, highly graphitized carbon fibers, which are coated by N-doped graphitized carbon (GF-FeO@NGC). Numerous studies have demonstrated the favorable specific capacity and cycling stability of these composite electrodes, highlighting their promising potential for practical applications.

The interlayer space of graphite presents a novel option for loading Fe_2_O_3_. Notably, the repeated insertion of Li^+^ causes an expansion in the layer spacing of SG, weakening the Van der Waals force between layers, which makes the intercalation easier. Moreover, due to the excellent microwave absorbing and conductive properties of graphite, the efficient coupling of microwave radiation with graphite facilitates the rapid removal of impurities and intercalants. Microwave radiation on SG induces a Joule heat–discharge–plasma coupled effect, resulting in rapid heating characterized by thermal shock. This causes the impurities and intercalants to be quickly released, leading to further expansion of the layer spacing and forming microwave-puffed graphite (MPG). Fe_2_O_3_-MPG intercalation compound (Fe_2_O_3_-MPGIC) can be fabricated by integrating Fe_2_O_3_ into the layers of MPG, which can significantly improve the electrical conductivity of Fe_2_O_3_ and mitigate issues arising from volume expansion [12], such as inadequate mechanical stability and detachment of copper foil.

However, the strong agglomeration tendency and thermal stability of Fe_2_O_3_ particles present challenges when attempting to embed it directly into graphite layers. The potential of graphite frameworks can be fully realized by embedding appropriate precursors into graphite interlayers and subsequently facilitating their growth into Fe_2_O_3_ particles. The embedding of metal chlorides, especially FeCl_3_, into graphite interlayers as precursors has attracted considerable interest [12,13,14]. Notably, the graphite framework can be rapidly intercalated with FeCl_3_ to form GICs since the microwave-induced Joule heat can enhance the internal energy of gaseous reactant molecules and strengthen the kinetics of intercalation reaction [15].

In this paper, a microwave-assisted method for recovering spent graphite to construct a high-performance Fe_2_O_3_-MPGIC anode with a sandwich structure is proposed. The method utilizes microwave-induced thermal shock to instantaneously release interlayer impurities and intercalating agents, leading to an increase in interlayer spacing and the formation of a three-dimensional framework. FeCl_3_ infiltrates the interlayer of the MPG through microwave-induced gasification intercalation and is converted in situ to Fe_2_O_3_ particles during heat treatment. The Fe_2_O_3_-MPGIC with a sandwich structure not only maintains the stability and electrical conductivity of graphite but also exhibits enhanced electrochemical properties. This innovative approach offers an efficient way to process spent graphite and fabricate anodes for high-capacity lithium storage.

## 2. Results and Discussion

### 2.1. Characterization

**Figure 1:**
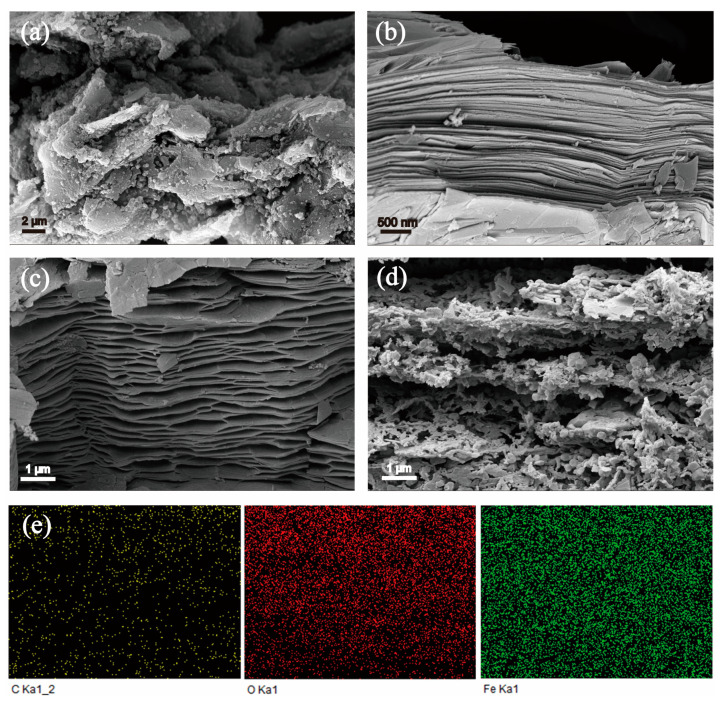
SEM of (**a**) SG, (**b**) MPG, (**c**) FeCl_3_-MPGIC, and (**d**) Fe_2_O_3_-MPGIC as well as (**e**) EDS of Fe_2_O_3_-MPGIC.

The microstructure of the samples was clearly visualized using SEM. As illustrated in Figure 1a, the SG exhibits clumping due to the presence of residual polymer binders. The surface of the SG is characterized by numerous aggregated particles, predominantly composed of residual binders, derived products of SEI, and decomposition products of the electrolyte [16,17]. MPG exhibits an obvious layered structure and expanded layer spacing in Figure 1b, which is attributed to the instant release of impurities and intercalants in the interlayer by microwave thermal shock. As shown in Figure 1c, the FeCl_3_-MPGIC formed by microwave irradiation after mixing MPG with FeCl_3_ has a larger layer spacing which is the result of the diffusion of iron chlorides, such as [FeCl_4_]^−^, into the interlayer. In addition, numerous small particles are uniformly distributed in the interlayer of the MPG framework, which are the iron chlorides deposited on the MPG framework after cooling. After heat treatment, these iron chlorides gradually convert to Fe_2_O_3_ and agglomerate into particles that are larger in size compared to the iron chlorides.

The significant increase in particle size of Fe_2_O_3_ causes a further expansion of the layer spacing of MPG, so that the Fe_2_O_3_-MPGIC exhibits a distinct sandwich structure, as shown in Figure 1d. Fe_2_O_3_ particles in Fe_2_O_3_-MPGIC are widely and uniformly distributed within the interlayers of the MPG framework, which can restrict the volume expansion of Fe_2_O_3_ particles during the lithiation process, thereby ensuring the stability of the interface between Fe_2_O_3_ particles and the electrolyte. In addition, a large number of pores were etched by Fe^3+^ on the MPG framework during the heat treatment. The enhanced pore structure facilitates the infiltration and permeation of the electrolyte and promotes charge transfer on the Fe_2_O_3_ particles in the MPG. Figure 1e presents an energy dispersive spectroscopy (EDS) elemental map that vividly illustrates the distribution of carbon (C), oxygen (O), and iron (Fe) across the sample. The uniform dispersion of these elements is indicative of the Fe_2_O_3_ particles being homogeneously integrated within the MPG framework., confirming the successful creation of Fe_2_O_3_-MPGIC composite materials.

**Figure 2:**
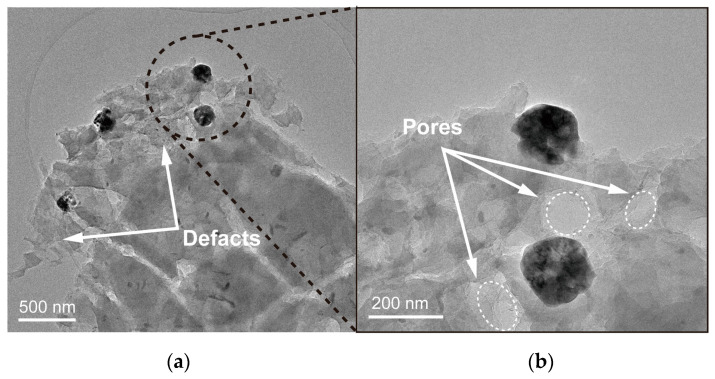
TEM of (**a**) defects and (**b**) pore structure of Fe_2_O_3_-MPGIC.

TEM was conducted for the in-depth research on the pore structure and surface defects in Fe_2_O_3_-MPGIC. As depicted in Figure 2, the Fe_2_O_3_-MPGIC structure is characterized by uniformly sized Fe_2_O_3_ particles that are firmly anchored to graphite sheets. This observation suggests that ferric chloride, during the intercalation process, is evenly dispersed within the interlayers of FeCl_3_-MPGIC and subsequently aggregates and converts into Fe_2_O_3_ particles upon heat treatment, resulting in a consistent particle distribution. In addition, visible surface defects (Figure 2a) and pore structure (Figure 2b) can be observed on the graphite sheet. The TEM results further demonstrate that the MPG framework is strongly etched by Fe^3+^ during the thermal treatment, resulting in the formation of abundant pore structures and surface defects. This will help increase the contact between the Fe_2_O_3_-MPGIC electrode and the electrolyte, thereby improving the Li ions and electrons transport efficiency.

**Figure 3:**
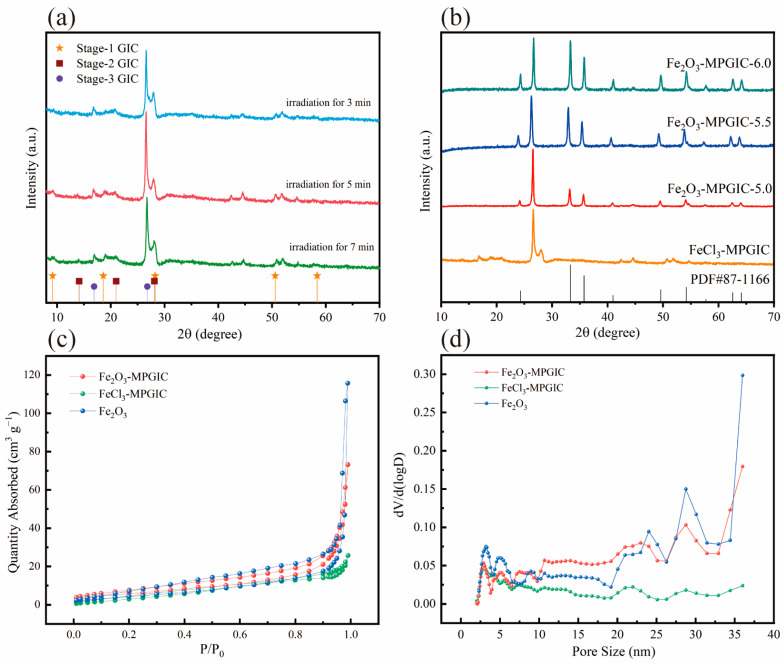
XRD patterns of (**a**) FeCl_3_-MPGIC and (**b**) Fe_2_O_3_-MPGIC, and BET results (**c**,**d**) of Fe_2_O_3_, FeCl_3_-MPGIC and Fe_2_O_3_-MPGIC.

The crystal structure of the obtained samples was studied through XRD. In general, GICs with a layer of intercalators every *n* layers of graphite are called stage-*n* GICs. According to the diffraction peaks, it can be concluded that FeCl_3_ has been embedded into the graphite layers after microwave irradiation, and the FeCl_3_-MPGIC exhibits a typical GIC structure. As shown in Figure 3a, obvious diffraction peaks are observed at 9.18°, 18.6°, 28.24°, and 50.58° in the FeCl_3_-MPGIC sample, corresponding to the stage-1 intercalation structure of FeCl_3_ with graphite [18]. And the diffraction peaks at 14.1° and 21° correspond to stage-2, while the diffraction peak at 16.9° corresponds to the stage-3 intercalation structures [19].

In addition, the effect of microwave irradiation on the sample structure was analyzed by comparing the XRD patterns of samples obtained at different irradiation times. Compared to FeCl_3_-MPGIC irradiated for 3 min, the diffraction peaks located at 9.18°, 14.1°, and 18.6° are more pronounced in samples irradiated for more than 5 min. This indicates that after 5 min of irradiation, the samples obtained have a higher degree of intercalation structure. For the FeCl_3_-MPGIC irradiated for 7 min, the intensity of the diffraction peak located at 14.1° diminishes, which may be attributed to the high temperature caused by prolonged irradiation inhibiting the kinetics of FeCl_3_ intercalation into the graphite layers, thus inhibiting the formation of second-order intercalation structures. Therefore, 5 min of irradiation achieves the optimal intercalation structure of FeCl_3_-MPGIC with the shortest irradiation time and minimum energy consumption. 

Further analysis was conducted on the crystal structure of Fe_2_O_3_-MPGIC to reveal the effect of the mass ratio between FeCl_3_ and MPG. As illustrated in Figure 3b, the Fe_2_O_3_-MPGIC samples exhibit obvious diffraction peaks at 24.15°, 33.16°, 35.63°, 40.86°, 49.46°, 54.07°, 62.44°, and 64°, respectively, corresponding to the (0 1 2), (1 0 4), (1 1 0), (1 1 3), (0 2 4), (1 1 6), and (2 1 4) crystal planes of Fe_2_O_3_, which are consistent with the standard diffraction peaks in PDF # 87-1166. This indicates that the iron chloride such as [FeCl_4_]^−^ in the interlayer of FeCl_3_-MPGIC can be converted to Fe_2_O_3_ after heat treatment. The intensities of the Fe_2_O_3_ characteristic peaks in the XRD pattern of Fe_2_O_3_-MPGIC increased with the rise in the proportion of ferric chloride. This increase demonstrated that Fe_2_O_3_-MPGIC with various loadings was synthesized by mixing MPG with ferric chloride in different ratios. In addition, compared to the diffraction peak of FeCl_3_-MPGIC, Fe_2_O_3_-MPGIC has almost no corresponding peaks of intercalation structure, which is mainly attributed to the significant increase in layer spacing caused by the aggregation of iron chloride and the growth of Fe_2_O_3_ during the conversion process, destroying the original form of crystal planes.

N_2_-adsorption desorption testing was performed to characterize the changes in the pore structure of Fe_2_O_3_-MPGIC and further investigate its surface characteristics. Figure 3c shows the isothermal curves of Fe_2_O_3_, FeCl_3_-MPGIC and Fe_2_O_3_-MPGIC. Fe_2_O_3_-MPGIC has a higher adsorption capacity compared to Fe_2_O_3_ and FeCl_3_-MPGIC (0.1 < P/P_0_ < 0.3). This can be attributed to the expansion of the layer spacing of the MPG framework during the formation of Fe_2_O_3_ particles and the pore structure etched by Fe^3+^ during thermal treatment. The increased adsorption capacity also results in an increase in the specific surface area. As depicted in Table 1, the specific surface area of Fe_2_O_3_-MPGIC has been increased due to the growth and accumulation of Fe_2_O_3_ particle, coupled with the additional expansion of the MPG interlayers during the synthesis process. In addition, the changes in the specific surface area of Fe_2_O_3_-MPGIC under different material ratios are further analyzed. With an increase in the mass ratio of FeCl_3_, the specific surface area of Fe_2_O_3_-MPGIC significantly decreases. This is primarily due to the increased presence of Fe^3+^, which enhances the etching effect on the MPG framework surface during thermal treatment. Therefore, Fe_2_O_3_-MPGIC-6.0, with a lower specific surface area, exhibits a more abundant pore structure and more prominent surface defects.

The pore size distribution of the Fe_2_O_3_-MPGIC surface is analyzed to further study its pore structure. As depicted in Figure 3d, the pore structure of Fe_2_O_3_-MPGIC remains relatively consistent at small pore sizes (≤3 nm) in comparison to FeCl_3_-MPGIC. However, the amount of pore structure increases significantly for pores larger than 7 nm, particularly for pores larger than 20 nm, which is the predominant form of the Fe_2_O_3_-MPGIC pore structure. This suggests that the inclusion of Fe_2_O_3_-MPGIC enriches the pore structure of the samples, resulting in a broader distribution of pore sizes in the composite material. Rich mesopores (2~50 nm) and the larger surface area can provide ion transport channels, and reduce diffusion resistance [20]. Therefore, Fe_2_O_3_-MPGIC demonstrates the potential as a lithium storage material due to its excellent structural characteristics.

**Figure 4:**
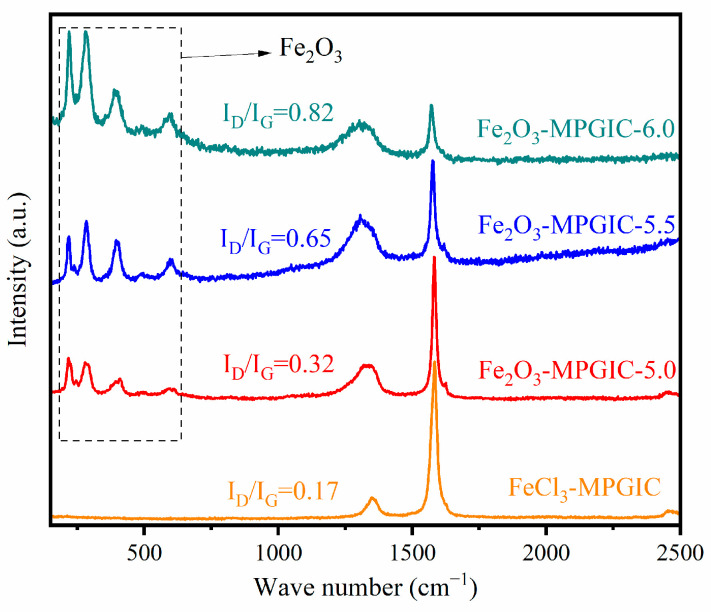
Raman spectra of FeCl_3_-MPGIC and Fe_2_O_3_-MPGIC.

The Fe_2_O_3_-MPGIC not only has a rich pore structure but also exhibits various defects. The Raman spectrum is utilized to investigate the defects and characteristics of the carbon structure in Fe_2_O_3_-MPGIC. As shown in Figure 4, Fe_2_O_3_-MPGIC and FeCl_3_-MPGIC display distinct peaks at 1360 and 1590 cm^−1^, which corresponds to the D and G bands, respectively, characterizing the carbon structure of the material. The crystallinity of the carbon materials in the samples can be compared by calculating the ID/IG values, which correspond to the disordered carbon structure (D band) and the graphitized carbon structure (G band). The I_D_/I_G_ value of FeCl_3_-MPGIC is 0.17, indicating a high degree of graphitization and minimal structural defects in the carbon structure of the material. The I_D_/I_G_ values of the Fe_2_O_3_-MPGIC increase gradually with the increase in mass ratio, reaching 0.32, 0.65, and 0.82, respectively. This indicates that the etching effect of Fe^3+^ on the MPG framework becomes more pronounced as the volume of FeCl_3_ increases. It indicates that the structural defects of Fe_2_O_3_-MPGIC significantly increase with the increase in mass ratio, which aligns with the presence of various defects observed in the microstructure of Fe_2_O_3_-MPGIC in the SEM results.

Moreover, Fe_2_O_3_-MPGIC exhibits significant peaks at 215, 275, 390, and 590 cm^−1^, which correspond to the Fe-O bonds in the Fe_2_O_3_ structure [21]. As the mass ratio increases, the peak intensity also rises. On one hand, the increase in Fe_2_O_3_ content leads to an enhancement of the vibration signal of the Fe-O bond, On the other hand, the Fe_2_O_3_-MPGIC framework contains numerous structural defects, which expose more Fe_2_O_3_ particles and result in more significant molecular signals. However, few peaks are observed at 200~600 cm^−1^ in FeCl_3_-MPGIC, mainly due to the substantial amount of FeCl_3_ within the crystal structure of MPG, which is concealed by the complete graphite layer. As for Fe_2_O_3_-MPGIC, the increased structural disorder means the formation of numerous structural defects in the composite material. The presence of structural defects exposes more Fe_2_O_3_ particles and facilitates the infiltration of electrolyte. Therefore, Fe_2_O_3_-MPGIC exposes more active sites for Li^+^ reactions, additionally increasing the lithium storage capacity of the Fe_2_O_3_-MPGIC electrode.

**Figure 5:**
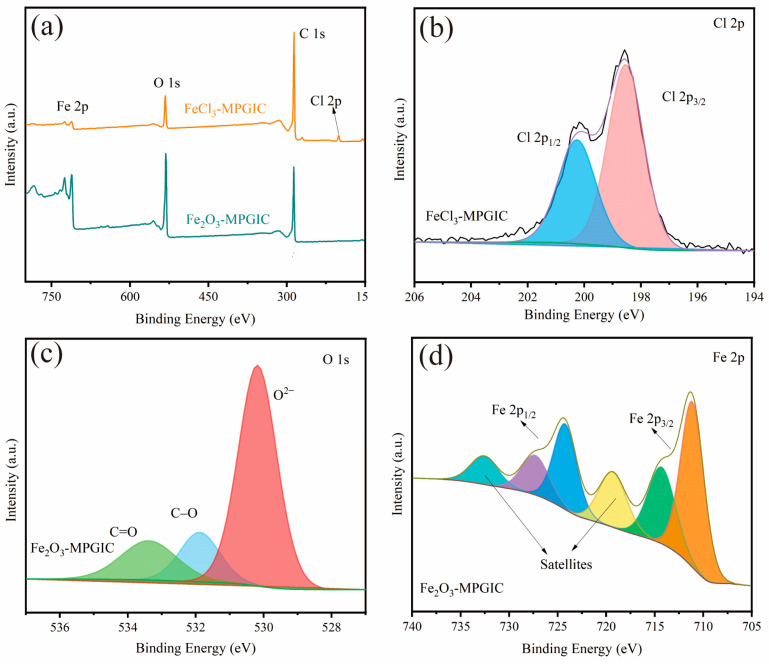
XPS spectra: (**a**) survey of FeCl_3_-MPGIC and Fe_2_O_3_-MPGIC, (**b**) Cl 2p of FeCl_3_-MPGIC, (**c**) O 1s and (**d**) Fe 2p of Fe_2_O_3_-MPGIC.

XPS was performed to further study the elemental composition and chemical properties of Fe_2_O_3_-MPGIC. As depicted in Figure 5a, the XPS survey spectrum of FeCl_3_-MPGIC reveals the presence of notable amounts of C, Cl, and Fe elements, confirming the presence of iron chlorides such as [FeCl_4_]^−^ in the MPG framework. The presence of a small amount of the O element is attributed to the sample’s affinity for atmospheric oxygen and moisture. The Fe_2_O_3_-MPGIC clearly contains C, O, and Fe elements, with almost no characteristic peaks of the Cl element, indicating that almost all iron chlorides are converted to Fe_2_O_3_ after thermal treatment. Furthermore, the more defined O 1s peak observed in the Fe_2_O_3_-MPGIC spectrum provides compelling evidence that Fe^3+^ ions, having released their chloride components, have combined with oxygen to form the Fe_2_O_3_ phase.

Further analysis of the spectra of each element verifies the form of existence of the corresponding components. As shown in Figure 5b, there are two obvious fitting peaks in the Cl 2p map of FeCl_3_-MPGIC at 198.5 eV and 200.3 eV, which can be identified as Cl 2p_3/2_ and Cl 2p_1/2_ of chlorides, respectively. It indicates that the iron chlorides embedded in the MPG framework maintain a binding energy close to the crystalline state [14]. An in-depth analysis of the O1s spectrum of Fe_2_O_3_-MPGIC depicted in Figure 5c is conducted in order to verify the presence of Fe_2_O_3_. The fitted peaks located at 530.2, 531.9, and 533.4 eV correspond to the presence of three distinct chemical structures of O^2−^, C-O, and C=O, respectively [22,23]. The significant O^2−^ fitting peak suggests the extensive binding forms of Fe^3+^ and O^2−^, indicating the presence of Fe_2_O_3_.

As depicted in Figure 5d, the Fe 2p spectrum of Fe_2_O_3_-MPGIC exhibits two distinct peaks at 711.3 eV and 724.5 eV, corresponding to Fe 2p3/2 and Fe 2p1/2, respectively, which proves that an iron state exists in the form of trivalent Fe^3+^. Additionally, two satellite peaks are observed at 719.5 eV and 732.7 eV, confirming the widespread presence of Fe_2_O_3_ [10]. Therefore, through XPS analysis, it was found that the iron chlorides in FeCl_3_-MPGIC are widely present in the interlayer of the MPG framework. After thermal treatment, almost all the iron chlorides in the interlayer are converted into Fe_2_O_3_, thus constructing a sandwich structure of Fe_2_O_3_-MPGIC.

### 2.2. Electrochemical Performance

**Figure 6:**
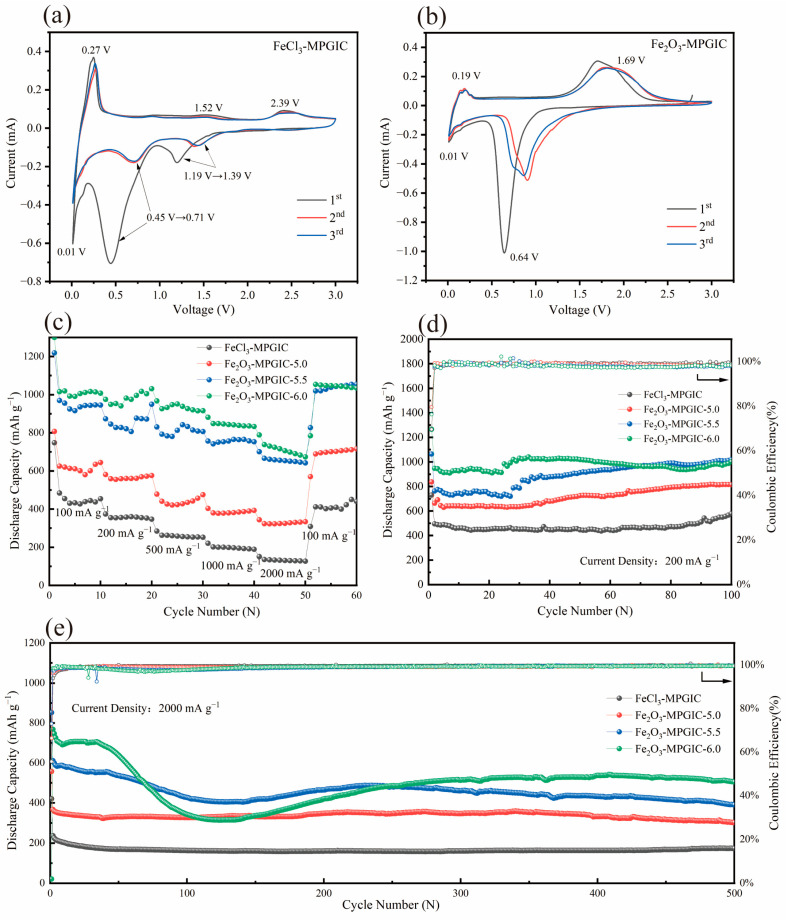
CV curves of (**a**) FeCl_3_-MPGIC and (**b**) Fe_2_O_3_-MPGIC, (**c**) cycling performance at 200 mA g^−1^, (**d**) rate performance, and (**e**) long-cycle performance at 2000 mA g^−1^.

The electrochemical performance of the FeCl_3_-MPGIC and Fe_2_O_3_-MPGIC electrodes for LIBs was evaluated by assembling coin-type half cells. The cyclic voltammetry (CV) tests were initially conducted at a scan rate of 0.1 mV s^−1^ within the voltage range of 0.01~3.0 V. Figure 6a shows the CV curve of FeCl_3_-MPGIC electrode, with prominent peaks observed at 1.19, 0.45, and 0.01 V during the cathodic scanning of the first cycle. Among them, the first two peaks correspond to a multi-step lithiation process in which Li^+^ are intercalated into the graphite interlayer and react with iron chlorides such as [FeCl_4_]^−^ within the interlayer. This process is highly irreversible, resulting in the cathodic peaks shifting from 1.19 V and 0.45 V to 1.39 V and 0.71 V, respectively, in subsequent cycles [14]. The cathodic peak located at 0.01 V corresponds to the process of embedding Li^+^ into the graphite layer to form LiC_6_, indicating that the MPG framework can contribute additional capacity for lithium storage. During the anode scanning phase, the peak located at 0.27 V corresponds to the decomposition of LiC_6_ embedded in the interlayer and the removal of Li^+^. Subsequently, the reverse process of lithium intercalation occurs at 1.52 V and 2.39 V, corresponding to 1.19 V and 0.45 V, respectively. After the highly irreversible reaction of lithium intercalation during the first cycle, the anodic scanning stage exhibits good reversibility. The analysis of the current intensity in the CV curve indicates that the FeCl_3_-MPGIC electrode, which contains iron chlorides in the interlayer, has a lithium storage capacity comparable to that of the MPG framework.

After thermal treatment, the iron chlorides in the interlayer transformed into Fe_2_O_3_, resulting in significant variations in their capacity for lithium storage. As shown in Figure 6b, the CV curve of the Fe_2_O_3_-MPGIC electrode exhibits notable changes in both peak voltage and peak current. The cathodic peak corresponding to the reaction between Fe_2_O_3_ and Li^+^ to form Fe^0^ is located at 0.64 V, which is significantly different from the lithium storage potential of iron chlorides. During the anodic scan, the peak located at 1.69 V corresponds to the oxidation process of Fe^0^ converting to Fe^3+^. Similar to the FeCl_3_-MPGIC electrode, the MPG framework also contributes a portion of lithium storage capacity to Fe_2_O_3_-MPGIC, as indicated by the peaks located at 0.01 V and 0.19 V in the CV curve. Comparing the CV curves of FeCl_3_-MPGIC and Fe_2_O_3_-MPGIC, it is evident that the Fe_2_O_3_-MPGIC electrode exhibits a higher peak current. This suggests that Fe_2_O_3_ plays a significant role in the lithium storage capacity of the composite electrode, indicating that Fe_2_O_3_ dominates the overall lithium storage capacity.

The cycling performance of Fe_2_O_3_-MPGIC electrodes was evaluated through galvanostatic charge/discharge testing at a current density of 200 mA g^−1^. As depicted in Figure 6c, the Fe_2_O_3_-MPGIC electrode exhibited superior cycling performance and lithium storage capacity compared to the FeCl_3_-MPGIC electrode. With an increase in the mass ratio, the reversible capacity of the electrode significantly improved, indicating that a higher mass results in a larger volume of iron chlorides in the interlayer, thereby allowing more Fe_2_O_3_ particles to be loaded into the interlayer of the MPG framework after thermal treatment. Specifically, the initial discharge capacity of Fe_2_O_3_-MPGIC-6.0 is 1265.6 mAh g^−1^, with an initial coulombic efficiency is 76.2%. The significant irreversibility is mainly attributed to the irreversible loss of active Li^+^ caused by the formation of SEI. The capacity of the electrode slowly increases during the cycling process, indicating that the electrode becomes more thoroughly wetted in the electrolyte and is further activated as the cycling continues [24].

After 100 cycles, the Fe_2_O_3_-MPGIC-6.0 electrode maintains a high reversible capacity of 1000.6 mAh g^−1^, significantly surpassing the 578.3 mAh g^−1^ capacity of FeCl_3_-MPGIC, which demonstrates the excellent lithium storage capacity and cycling stability of the Fe_2_O_3_-MPGIC-6.0 electrode. The outstanding cycling performance is primarily attributed to the excellent electrical conductivity provided by the MPG framework and the mitigated volume expansion of Fe_2_O_3_ through the sandwich structure. In addition, the MPG framework etched by Fe^3+^ possesses a rich pore structure and surface defects, which can promote the electrolyte infiltration and Li^+^ diffusion. Therefore, the sandwich structure of the Fe_2_O_3_-MPGIC electrode improves electron and Li^+^ transport, enhancing the electrical contact and resulting in higher reversible capacity and excellent cycling stability.

Furthermore, the rate performance of the electrodes was compared within the current density range of 100 mA g^−1^ to 2000 mA g^−1^. As shown in Figure 6d, Fe_2_O_3_-MPGIC-6.0 exhibits excellent rate performance, maintaining high reversible capacities of 1007.7, 1031.2, 915.9, 833, and 673.5 mAh g^−1^ at 100, 200, 500, 1000, and 2000 mA g^−1^, respectively. The outstanding rate performance is not only 4–5 times higher than the capacity of FeCl_3_-MPGIC electrode at high current densities, but also up to 10 times higher than that of commercial graphite (62.5 mAh g^−1^ at 2000 mA g^−1^). When the current density returns to 100 mA g^−1^, the capacity of Fe_2_O_3_-MPGIC-6.0 recovers to 1034 mAh g^−1^, significantly higher than the capacity of FeCl_3_-MPGIC electrodes (440 mAh g^−1^) and commercial graphite (369 mAh g^−1^).

Notably, the excellent rate performance of Fe_2_O_3_-MPGIC-6.0 draws support from the sandwich structure constructed by the MPG framework to provide reliable mechanical strength for the electrode, so as to ensure that excellent structural stability can be maintained under repeated lithiation and de-lithiation, especially at high current densities. Furthermore, the etching of Fe^3+^ on the MPG framework during the thermal treatment greatly enriches the pore structure and surface defects of the electrode, providing a shorter diffusion path for Li^+^ and ensuring efficient charge transfer at high rates. As a result, the electrode exhibits a high reversible capacity, even at high current density. In addition, the excellent rate performance may also be contributed by the pseudo-capacitance on the electrode surface, which can promote rapid electrochemical reactions on the electrode surface [25].

**Figure 7:**
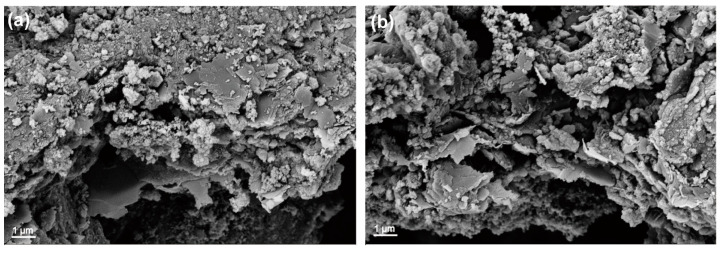
SEM of the anode (**a**) after 150 cycles and (**b**) after 400 cycles at 2000 mA g^−1^.

Considering the excellent cycling and rate performance of the Fe_2_O_3_-MPGIC electrode, the long-term cycling performance of the electrode was further tested at a current density of 2000 mA g^−1^ to study the cycling stability. As shown in Figure 6e, at 2000 mA g^−1^, Fe_2_O_3_-MPGIC-6.0 still exhibited excellent cycling stability after 500 cycles, maintaining a high reversible capacity of 504.4 mAh g^−1^, and the coulombic efficiency remains close to 100% throughout the cycling period. In contrast, the FeCl_3_-MPGIC electrode exhibited a reversible capacity of only 173.1 mAh g^−1^. This demonstrates that the sandwich structure can effectively alleviate the volume expansion of Fe_2_O_3_ particles, thus ensuring the structural stability of the electrode. The rich pore structure and surface defects on the MPG framework also enhance the diffusion of Li^+^ and facilitate charge transfer, allowing the electrode to maintain a high reversible capacity while exhibiting excellent cycling stability. However, the long cycle performance curve of Fe_2_O_3_-MPGIC-6.0 shows a decreasing and then an increasing trend. Upon careful comparison, a similar trend is observed for Fe_2_O_3_-MPGIC-5.5. This phenomenon may be attributed to the increased loading of Fe_2_O_3_, which stresses the structural stability of the MPG framework during high rate cycling without activation. While increased loads enhances specific capacity, it may simultaneously diminish the strength of composite materials [26]. Remarkably, as the charge/discharge cycling continues, the material appears to gradually adapt to this structural change, thereby recovering some of its electrochemical properties. This adaptation is corroborated in Figure 7, where the MPG framework still confines most of the Fe_2_O_3_ particles even after 400 cycles at 2000 mA g^−1^.

**Figure 8:**
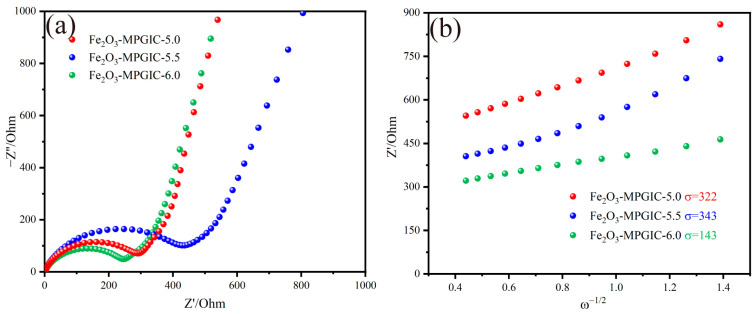
(**a**) EIS spectra and (**b**) the linear relation between Z′ and ω^−1/2^ of FeCl_3_-MPGIC and Fe_2_O_3_-MPGIC.

EIS was conducted to analyze the electrochemical kinetic behavior of Fe_2_O_3_-MPGIC electrode. As shown in Figure 8a, the spectrum mainly includes a semicircle at medium and high frequency and a linear track at low frequency. The intercept between the line and the horizontal axis in the high-frequency region corresponds to the electrolyte related impedance (*R*_S_), the semicircle diameter corresponds to the charge transfer impedance (*R*_CT_), and the linear trajectory in the low-frequency region corresponds to the Weber impedance (*Z*_W_), which can reflect the diffusion ability of Li^+^ of the electrode [27]. Z-view software is employed to fit and analyze the curves. A solution resistance (*R*_S_), charge transfer resistance (*R*_CT_), and Weber impedance (*W*_O_) constitute the equivalent circuit together which is connected in parallel with a constant phase element (CPE). Table 2 shows the fitted *R*_S_ and *R*_CT_ values. The *R*_S_ of Fe_2_O_3_-MPGIC is basically the same, indicating that the impedance of the electrode to the electrolyte is basically the same. The *R*_CT_ value of the electrode shows a trend of increasing and then decreasing with the increase in mass ratio. The increase in *R*_CT_ may be attributed to the aggregation of Fe_2_O_3_ particles which disrupts the layered structure of the MPG framework, thereby weakening the efficiency of charge transfer. The subsequent decrease in impedance is caused by the increase in pore structure and surface defects on the MPG framework, which facilitate the infiltration of electrolyte and enhance the charge transfer. The above results indicate that the rich pore structure and surface defects play an important role in reducing the internal resistance of batteries and promoting charge transfer.

Furthermore, the following equation is used to fit the curve in the low-frequency region to characterize the Li^+^ diffusion ability of the electrode:

(1) $$D=R^{2}T^{2}/2A^{2}n^{4}F^{4}C^{2}\sigma^{2}$$

(2) $$Z^{\prime }={\;R}_{S}+{\;R}_{\mathrm{CT}}+\sigma \omega^{-1/2}$$

where *R* is the gas constant, *T* represents the Kelvin temperature, usually taken as 298 K, *A* indicates the surface area of the electrode, *n* is the number of charge transfers during the redox process, *F* denotes the Faraday constant, *C* depicts the concentration of lithium ions, and *σ* symbolizes the Weber coefficient. By fitting the linear relationship between *Z*′ and *ω*^−1/2^ (*ω* is the angular frequency), the value of *σ* can be obtained [28].

The value of *σ*^2^ is inversely proportional to the diffusion coefficient *D*, where lower values *σ* corresponding to a larger Li^+^ diffusion coefficient, reflecting a stronger Li^+^ diffusion ability. As shown in Figure 8b, similar to the impedance results fitting from the medium and high frequency, the Li^+^ diffusion ability of Fe_2_O_3_-MPGIC electrode also first decreases and then increases. This is mainly attributed to the increase in the mass ratio weakening the promotion of graphite on Li^+^ diffusion, resulting in a reduced Li^+^ diffusion ability. As the mass ratio increases, the etching effect of Fe^3+^ on the MPG framework is enhanced. The rich pore structure and surface defects promote electrolyte infiltration, which shortens the transfer path of Li^+^ and enhances its diffusion ability. The EIS results indicate that the impedance of the Fe_2_O_3_-MPGIC electrode is generally at a lower level due to the excellent conductivity provided by the MPG framework. Moreover, the rich pore structure and surface defects on MPG facilitate the infiltration of electrolytes, further enhancing the diffusion of Li^+^ and charge transfer efficiency.

**Figure 9:**
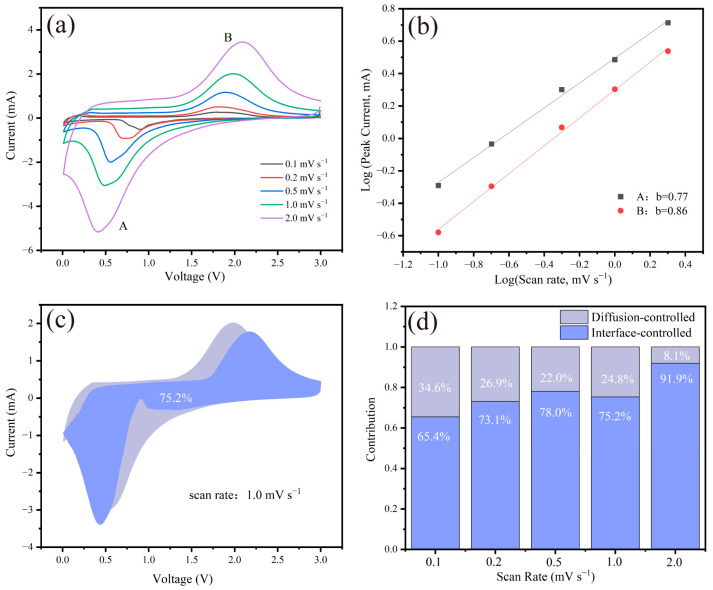
(**a**) CV curves of Fe_2_O_3_-MPGIC at scan rates ranging from 0.1 to 2.0 mV s^−1^. (**b**) The fitted linear relationship between log *i* and log *v*. (**c**) Pseudo-capacitance contribution at a scan rate of 1.0 mV s^−1^. (**d**) The ratio of pseudo-capacitance contribution of the Fe_2_O_3_-MPGIC electrode at various scan rates.

In order to study the pseudo-capacitance effect of Fe_2_O_3_-MPGIC-6.0 electrode, CV tests were conducted at scan rates ranging from 0.1 to 2.0 mV s^−1^. Figure 9a shows the CV curves of the electrodes at 0.1, 0.2, 0.5, 1.0, and 2.0 mV s^−1^. The relationship between peak current (*i*) and scanning rate (*v*) follows the equation

(3) $$i=av^{b}$$

where *i* and *v* refer to the peak current and scanning rate, respectively, and *a* and *b* are constants. The value of *b*, determined by the linear fitting slope of log *v*-log *I*, ranges from 0.5 to 1.0, where 0.5 indicates the battery process controlled by diffusion effect and 1.0 represents the capacitive process controlled by surface effect [29]. As shown in Figure 9b, the *b* values of the cathode peak (A) and anode peak (B) of Fe_2_O_3_-MPGIC-6.0 are 0.77 and 0.86, respectively, indicating that the reaction current is an interaction between battery process and capacitive process. This suggests that the Fe_2_O_3_-MPGIC-6.0 electrode is affected by the pseudo-capacitance effect during the cycling process. Based on the following equation, the contribution of pseudo-capacitance is further quantitatively analyzed:

(4) $$i=k_{1}v\;+k_{2}v^{1/2}$$

where *k*_1_, *k*_2_ are the constant [30]. The reaction current is divided into pseudo-capacitance contribution (*k*_1_*v*) and diffusion process contribution (*k*_2_*v*^1/2^), where the value of *k*_1_ can be calculated through the linear relationship between *iv*^−1/2^ and *v*^1/2^. As shown in Figure 9c, when the scanning rate is 1.0 mV s^−1^, the pseudo-capacitance contribution of the Fe_2_O_3_-MPGIC-6.0 electrode is 75.2%. As the scanning rate increased from 0.1 to 2 mV s^−1^ (Figure 9d), the pseudo-capacitance contribution of Fe_2_O_3_-MPGIC-6.0 electrode increased from 65.4% to 91.9%. This indicates that the pseudo-capacitance effect has a significant contribution to the lithium storage capacity, especially promoting the storage process of Li^+^ during high-rate cycling. Combined with the excellent rate performance of Fe_2_O_3_-MPGIC-6.0 electrode, the sandwich structure provides structural stability while realizing enhanced charge transfer, and the remarkable pseudo-capacitance effect also effectively enhanced the lithium storage capacity of the electrode at a high current density.

**Figure 10:**
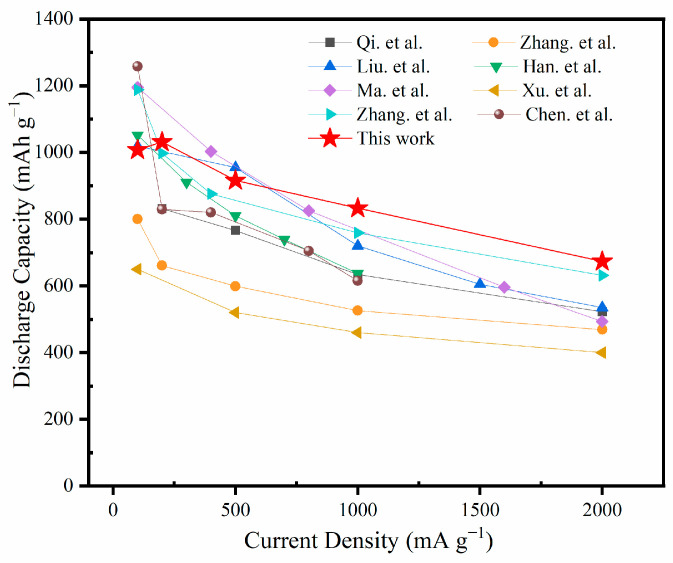
Comparison on lithium storage capacity between Fe_2_O_3_-MPGIC-6.0 electrode and other relevant studies [31,32,33,34,35,36,37,38].

Compared with the relevant studies on graphite-based Fe_2_O_3_ composite electrodes (Figure 10), the composite electrode in this study demonstrates superior rate capability while achieving a higher specific capacity. In summary, the obvious enhancement in the Li storage and cycling performance of Fe_2_O_3_-MPGIC electrodes can be attributed to the following reasons. (1) In Fe_2_O_3_-MPGIC electrodes, the MPG framework serves as a conductive network to accelerate electron transport, and the expanded interlayer spacing shortens the diffusion path of Li^+^, improving charge transfer on the composite electrode, thereby ensuring high reversible capacity and excellent rate performance of the electrode. (2) Owing to the support and restriction provided by the MPG framework on Fe_2_O_3_ particles, the volume expansion of Fe_2_O_3_ particles during the cycle can be alleviated, while ensuring the structural stability of the electrode. (3) The etching of the MPG by Fe^3+^ during thermal treatment significantly enriches the surface defects and pores of Fe_2_O_3_-MPGIC electrode. This leads to a shorter diffusion path for Li^+^, faster charge transfer, and an increase in lithium storage sites within the electrode, resulting in higher reversible capacity and excellent cycling performance. (4) The sandwich structure exhibits significant pseudo-capacitance, which ensures efficient charge transfer between electrodes even at high current densities, thereby improving the rate performance of Fe_2_O_3_-MPGIC electrodes.

## 3. Materials and Methods

### 3.1. Materials

Spent lithium-ion batteries were obtained from Shandong Jiuli Electronic Technology Co., Ltd., Zaozhuang, China. Potassium persulfate (K_2_S_2_O_8_) was obtained from Shanghai McLean Biochemical Co., Ltd., Shanghai, China. Phosphoric acid (H_3_PO_4_) was obtained from Sinopharm Chemical Reagent Co., Ltd., Shanghai, China. Iron trichloride (FeCl_3_) was purchased from Shanghai Aladdin Biochemical Technology Co., Ltd., Shanghai, China.

### 3.2. Acquisition of SG

The spent lithium batteries must be fully discharged prior to manual disassembly to prevent safety hazards. The disassembled cathode material was recycled and used for other research, while the anode material was immersed in a 1 mol L^−1^ KOH solution to separate it from the copper foil. Subsequently, the anode powder was rinsed with acid and ample deionized water to neutralize the pH and eliminate surface impurities. After drying and sieving, the SG was obtained with the copper foil nearly intact.

### 3.3. Preparation of MPG Framework

The synthesis of MPG has been implemented in previous work [39]. Firstly, microwave treatment was utilized to quickly and effectively remove residual impurities from the SG. The SG was placed in a customized industrial microwave oven and treated for 15 s per 15 s interval at a power of 1000 W for 5 min. Subsequently, oxidative intercalation of the SG after removing impurities was conducted under the synergistic effect of K_2_S_2_O_8_ and H_3_PO_4_. After centrifugation, washing, and drying, the successfully intercalated SG was obtained and then treated for 5 min using the microwave process mentioned earlier. Due to the rapid decomposition and vaporization of the intercalant between graphite layers at high temperatures, part of the interlayer spacing of the SG was expanded, thus successfully obtaining an MPG framework with a clear and complete sandwich structure.

### 3.4. Preparation of Fe_2_O_3_-MPGIC

Due to the deliquescence of FeCl_3_ in the air atmosphere, the sample preparation process needs to be carried out in a glove box filled with argon gas. Firstly, the MPG framework and FeCl_3_ were mixed by grinding them together in a mass ratio of 1:5.0, 1:5.5, and 1:6.0 in the glove box. Subsequently, the mixture is loaded into a custom quartz reactor. The quartz reactor was placed in a microwave oven and purged with Ar gas at a flow rate of 200 mL min^−1^ to ensure an anhydrous and inert atmosphere.

**Figure 11:**
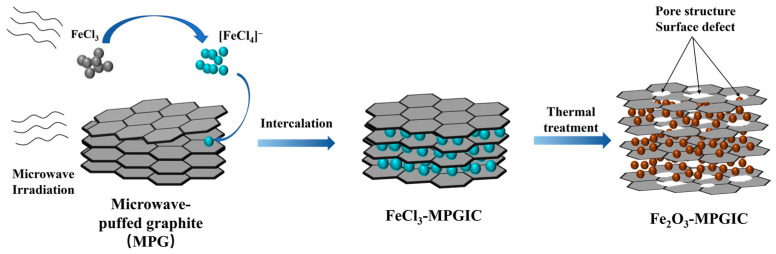
Schematic of synthesizing Fe_2_O_3_-MPGIC.

Intermittent microwave irradiation was used for the preparation of FeCl_3_-MPGIC. The detailed procedure is as follows. Firstly, the quartz reactor was irradiated at 900 W for 30 s, followed by intermittent irradiation (on: 9 s, off: 21 s) at 900 W for 3 to 7 min. Severe discharge phenomena can be observed in the reactor during this process. FeCl_3_ was excited into a gaseous mixture of Cl_2_ and ferric chloride, migrating toward the interlayer of the MPG framework. Intermittent irradiation can supply enough energy for the migration through excitation and discharge, while also preventing the direct decomposition of FeCl_3_ due to excessively high temperatures. Secondly, the FeCl_3_-MPGIC was obtained after multiple washings, vacuum filtration, and overnight drying. Lastly, Fe_2_O_3_-MPGIC was obtained by heating FeCl_3_-MPGIC to 550 °C at a rate of 5 °C min^−1^ and then holding it for 3 h in a muffle furnace, wherein the iron chloride embedded in the interlayer was in situ converted into Fe_2_O_3_. According to the difference in mass ratio, the obtained samples were recorded as Fe_2_O_3_-MPGIC-5.0, Fe_2_O_3_-MPGIC-5.5, and Fe_2_O_3_-MPGIC-6.0. The mechanism of synthesizing Fe_2_O_3_-MPGIC is primarily illustrated in Figure 11.

### 3.5. Characterization and Electrochemical Test

The morphology observation and microstructure measurements of as-prepared samples were carried out using scanning electron microscopy (SEM; SUPRA™55, ZEISS, Oberkochen, Germany) and transmission electron microscope (TEM; FEITalos F200x, FEI Company, Hillsboro, OR, USA). Moreover, the crystal structure was examined by X-ray diffraction (XRD; AERIS, PANalytical B.V., Almelo, The Netherlands) using Cu-K radiation, with 2θ values ranging from 8 to 70°. Furthermore, the carbon structure was investigated using Raman spectroscopy (HORIBA LabRAM HR Evolution, HORIBA Scientific, Paris, France) and the laser wavelength used in this experiment is 532 nm. X-ray photoelectron spectroscopy (XPS; Thermo Scientific K-Alpha, Thermo Fisher Scientific, Waltham, MA, USA) was employed to further analyze the chemical characteristics. In addition, the surface area and pore distribution of the sample were modeled by the N_2_ adsorption–desorption isotherm (Quanta Chrome Instruments Co., Ltd., Boyertown, PA, USA).

The electrochemical properties of the samples were analyzed by testing assembled CR2025 half-cells. At first, the active substance, super P carbon and sodium carboxymethyl cellulose (CMC) were mixed and ground in the mass ratio of 7:2:1 with deionized water as solvent. Then, the working electrode sheet was uniformly coated on the copper foil and vacuum dried at 60 °C for 12 h. The slurry thickness of the obtained electrode sheet was approximately 20 μm, and the mass loading of the active substance was calculated to be about 1.0 to 1.2 mg cm^−2^. When assembling the battery, the lithium metal sheet was used as the counter electrode, and the polyethylene porous composite film (Celgard 2400) was placed between it and the working electrode as the diaphragm. The electrolyte contained 1.0 mol L^−1^ LiPF_6_ in a solution of ethylene carbonate (EC) and dimethyl carbonate (DMC) (1:1 Vol%). The entire assembly process should be completed in a glove box under high purity Ar atmosphere (water and oxygen values not exceeding 0.1 ppm).

The galvanostatic charge/discharge performance test from 0.01 to 3 V at room temperature were performed on a LAND battery testing system (CT3001A, Wuhan Lanhe, Wuhan, China). Moreover, cyclic voltammetry (CV) was conducted on an electrochemical workstation (CHI 660e, Shanghai Chenhua, Shanghai, China), in which the test voltage was in the range of 0.01~3 V (versus Li/Li^+^), and the scan rate ranged from 0.1 mV s^−1^ to 2 mV s^−1^. In addition, electrochemical impedance spectroscopy (EIS) was carried out on the CHI 660e electrochemical workstation with a voltage amplitude of 10 mV and a frequency range of 100 kHz to 0.01 Hz. Z-view software (Zview2, Scribner Associates, Inc., Southern Pines, NC, USA) is employed to fit and analyze the curves.

## 4. Conclusions

This work not only develops green, economic, and high-performance anodes for LIBs, which have shown great potential in promoting the practical applications, but also provides a valuable reference for the resource disposal of SG, which is of great significance in the resource closed-loop management in the LIB industry. Fe_2_O_3_-MPGIC anode leverages the high theoretical specific capacity of Fe_2_O_3_ while maintaining the high electrical conductivity and structural stability of graphite. Additionally, the etching effect enriches the graphite layers with a porous structure, which provides more lithium-ion transport channels, enhancing the long cycles and rate performance of Fe_2_O_3_-MPGIC. In electrochemical tests, Fe_2_O_3_-MPGIC delivered a high reversible capacity of 1000.6 mAh g^−1^ at 200 mA g^−1^ after 100 cycles and exhibited excellent rate performance, retaining a capacity of 673 mAh g^−1^ when the current density was increased to 2000 mA g^−1^. It also presented obvious cycling stability, maintaining a capacity of 504.4 mAh g^−1^ at 2000 mA g^−1^ after 500 cycles. However, while increased Fe_2_O_3_ content can enhance the capacity, it may concurrently introduce challenges that could compromise the electrode’s long-term stability. Achieving an optimal balance between the mass fraction of Fe_2_O_3_ and the preservation of structural stability stands as a pivotal issue that must be addressed in the development of future electrodes of this category.

## Figures and Tables

**Table 1:** Specific surface area of samples.

Samples	Specific Surface Area (m^2^ g^−1^)
Fe_2_O_3_	17.408
MPG	13.267
FeCl_3_-MPGIC-6.0	17.335
Fe_2_O_3_-MPGIC-5.0	29.435
Fe_2_O_3_-MPGIC-5.5	24.067
Fe_2_O_3_-MPGIC-6.0	22.017

**Table 2:** *R*_S_ and *R*_CT_ of Fe_2_O_3_-MPGIC electrode.

Samples	*R*_S_	*R*_CT_
Fe_2_O_3_-MPGIC-5.0	1.626	300
Fe_2_O_3_-MPGIC-5.5	1.764	442.3
Fe_2_O_3_-MPGIC-6.0	1.749	246.3

## Data Availability

Data are contained within the article.
